# BAG5 Interacts with DJ-1 and Inhibits the Neuroprotective Effects of DJ-1 to Combat Mitochondrial Oxidative Damage

**DOI:** 10.1155/2017/5094934

**Published:** 2017-03-02

**Authors:** Li-xia Qin, Jie-qiong Tan, Hai-nan Zhang, Kousar Rizwana, Jia-hong Lu, Jian-guang Tang, Bo Jiang, Xiang-min Shen, Ji-feng Guo, Bei-sha Tang, Li-ming Tan, Chun-yu Wang

**Affiliations:** ^1^Department of Neurology, The Second Xiangya Hospital, Central South University, Changsha, China; ^2^The State Key Laboratory of Medical Genetics and School of Life Science, Central South University, Changsha, Hunan, China; ^3^Department of Biology, Allama Iqbal Open University, Islamabad, Pakistan; ^4^State Key Laboratory of Quality Research in Chinese Medicine, Institute of Chinese Medical Sciences, University of Macau, Macao, China; ^5^Department of Neurology, Xiangya Hospital, Central South University, Changsha, China; ^6^Key Laboratory of Hunan Province in Neurodegenerative Disorders, Central South University, Changsha, China; ^7^Neurodegenerative Disorders Research Center, Central South University, Changsha, China

## Abstract

Loss-of-function mutations in gene encoding DJ-1 contribute to the pathogenesis of autosomal recessive early-onset familial forms of Parkinson's disease (PD). DJ-1 is a multifunctional protein and plays a protective role against oxidative stress-induced mitochondrial damage and cell death, but the exact mechanism underlying this is not yet clearly understood. Here, using coimmunoprecipitation (Co-IP) and immunofluorescence methods, we prove that Bcl-2-associated athanogene 5 (BAG5), a BAG family member, interacts with DJ-1 in mammalian cells. Moreover, we show that BAG5 could decrease stability of DJ-1 and weaken its role in mitochondrial protection probably by influencing dimerization in stress condition. Our study reveals the relationship of BAG5 and DJ-1 suggesting a potential role for BAG5 in the pathogenesis of PD through its functional interactions with DJ-1.

## 1. Introduction

Parkinson's disease (PD) is a common neurodegenerative disorder characterized by progressive degeneration of dopaminergic neurons and the formation of Lewy bodies mainly in the substantia nigra pars compacta (SNpc) [1, 2]. Mitochondrial dysfunction and abnormal degradation of protein induced by environmental and genetic factors are assumed to be the major cause underlying Parkinson's pathogenesis. Until now, 18 genes responsible for Mendelian form of PD have been identified including *α*-synuclein* (PARK1/PARK4)* [3, 4], Parkin* (PARK2)* [5], PINK1* (PARK6)* [6], and DJ-1* (PARK7)* [7].

In 2003, Bonifati et al. found a large deletion and missense mutation in the* DJ-1 *gene in Italian and Dutch PD families, leading to identification of the* DJ-1 *gene for familial PD with autosomal recessive inheritance [7]. So far, 14 pathogenic mutations identified in* DJ-1* have been associated with PD. DJ-1 is a multifunctional protein playing a key role in transcriptional regulation, antioxidative stress reaction, and chaperone, protease, and mitochondrial regulation [8–12]. DJ-1 interacts with other proteins, such as Parkin, PINK1, and Hsp70, to protect cells against oxidative stress and maintain mitochondrial homeostasis [13, 14]. DJ-1 dysfunction thus leads to PD through impairing mitochondrial homeostasis, reducing the ability of antioxidation or inhibiting ubiquitin-proteasome pathway. However, the exact mechanism needs further elucidation.

The Bcl-2 associated athanogene (BAG) family plays potential role in neurodegenerative diseases [15, 16]. BAG family proteins act as adapters forming complexes with signaling molecules and molecular chaperones and take part in mounts of physiological processes, including stress signaling, cell death, and cell differentiation [17–19]. BAG5 contains multiple BAG domains. As a proapoptotic factor, BAG5 inhibits Hsp70 chaperone activity and Parkin E3 ubiquitin ligase activity and enhances dopaminergic neurodegeneration [15]. In addition, BAG5 can function as the nucleotide exchange factor of Hsp70 for the enhancement of protein refolding [20]. Recently, it has been found that BAG5 directly interacts with PINK1 and protects against mitochondrial oxidative damage through regulating PINK1 degradation [21]. However, the role of BAG5, as a chaperone, is far from being elucidated in oxidative stress.

Here, by Co-IP and immunofluorescence methods, we investigate whether BAG5 interacts with DJ-1 in mammalian cells. We further understand how BAG5 regulates DJ-1 levels and whether BAG5 exerts effect on DJ-1-mediated protective activity.

## 2. Materials and Methods

### 2.1. Expression Plasmids and siRNA

Full length BAG5 cDNA amplified from a human fetal brain library was cloned into the pEGFP-N1 vector and pcDNA3.1 vector (Clontech), respectively. Similarly, HA-DJ-1, DJ-1-GFP, and DJ-1-flag plasmids were constructed successfully as described previously [21]. Integrity of all constructs was confirmed by gene sequencing. The siRNA-Hsp70 duplex and scrambled siRNA were purchased from Santa Cruz Biotechnology (sc-29352).

### 2.2. Antibodies and Reagents

The antibodies against different tags and proteins used for immunoprecipitation and immunoblotting were as follows: GFP antibodies (rabbit polyclonal, ab290; mouse monoclonal ab1218, Abcam); DJ-1 antibodies (rabbit monoclonal, #5933, Cell Signaling; mouse monoclonal, ab11251, Abcam); mouse monoclonal Bag5 antibody (ab56738, Abcam); Myc antibodies (rabbit polyclonal, #2272, Cell Signaling; mouse monoclonal, #2276, Cell Signaling); HA-Tag rabbit monoclonal antibody (#3724, Cell Signaling); Hsp70 rabbit monoclonal antibody (#4876, Cell Signaling); mouse monoclonal ANTI-FLAG® M2 antibody (F1804, Sigma-Aldrich). Annexin V-FITC/PI Apoptosis Detection Kit was purchased from Thermo Scientific (V13242). Cycloheximide (R750107), rotenone (R8875), and rhodamine 123 (R8004) were purchased from Sigma.

### 2.3. Cell Culture Transfection and Stable Cell Line Generation

HEK293 cells were cultured in Dulbecco's modified Eagle's medium supplemented with 10% fetal bovine serum, 1% penicillin, and streptomycin at 37°C, 5% CO_2_ atmosphere. Cells were transfected with Lipofectamine 2000 (Invitrogen) according to the manufacturer's instructions. Further experiments were performed 24 h after transfection. Primary neuronal cultures were prepared from E17 rat primary hippocampal cells. Briefly, rat primary hippocampus was dissected in HBSS and digested with 0.25 mg/mL trypsin for 15 min at 37°C, followed by triturating through serial Pasteur pipettes with gradually decreased tip diameters. Trypsinized cells were plated at 150,000 cells/cm^2^ on glass coverslips precoated with Polylysine (50 *μ*g/mL). Cells were cultured with neurobasal medium supplemented with B27 supplement (2%), glutamax (500 *μ*M), and penicillin-streptomycin (50 *μ*g/mL) at 37°C, 5% CO_2_ atmosphere. At 4 DIV (days in vitro), the primary neurons were transfected by calcium phosphate method as previously described [22]. For stable expression cell lines, HEK293 cells were transfected with BAG5-Myc and DJ-1 HA or DJ-1-HA alone. After 48 hr transfection, cells were selected by 1.0 mg/mL G-418 antibiotic for 2 weeks.

### 2.4. Co-IP

Co-IP was performed as described previously [23]. After transfection, HEK293 cells were harvested in IP buffer (PBS containing 1% Triton X-100 and protease inhibitor cocktail). The lysate was centrifugated at 20,000 ×g for 30 min and aliquot of supernatant was used for Western blot analysis. The PVDF membrane, which contains transferred protein, was incubated with anti-HA antibody and pretreated protein G sepharose 4 fast for 2 hr, followed by rotating overnight at 4°C. Protein G sepharose complex was pelleted and washed three times with IP buffer. After being resolved by SDS-PAGE, immunoprecipitates were subjected to Western blot analysis.

### 2.5. Western Blot

Protein concentration was determined by BCA protein assay kit (Thermo Scientific) [24]. Before electrophoresis, samples were mixed with 2x SDS loading buffer thoroughly. Then, proteins were transferred to a PVDF membrane and the nonspecific binding was blocked by incubating the membrane in PBST (PBS containing 0.05% Tween 20) with 5% milk for 2 hr at room temperature. After that, the membrane was incubated with primary antibodies overnight at 4°C, followed by incubation with horseradish peroxidase-linked secondary antibody in PBST with 5% milk for 1 hr at room temperature. Bands were detected by enhanced chemiluminescence (ECL) reagent and exposure to X-Omat films. Densitometry analysis software was used to quantify.

### 2.6. Immunofluorescence

Immunofluorescence was performed using well established method [23]. Cells were transfected with plasmids using Lipofectamine 2000. After 24 hr, cells were fixed with PBS containing 4% w/v paraformaldehyde for 15 min. After washing three times, cells were permeabilized by incubation in 1x PBS containing 1% Triton X-100 for 1 hr and incubated with primary antibodies for 2 hr at room temperature. Appropriate secondary antibodies were used to detect the corresponding protein and cells were visualized under a laser confocal microscope (Leica). The colocalization coefficiency was analyzed with Image-Pro Plus 6.0 software following established method [25].

### 2.7. Mitochondrial Isolation

Cells were washed twice in 1x PBS and incubated for 30 min on ice in lysis buffer (68 mM sucrose, 200 mM mannitol, 50 mM KCl, 1 mM EDTA, 1 mM EGTA, and 1 mM dithiothreitol) with protease inhibitor cocktail. Cells were then lysed by 45 passages through a 25G 5/8 needle and centrifuged at 1,500 ×g for 10 min. Cytosolic extracts were recovered after centrifugation at 13,000 ×g for 20 min. The pellet contained crude mitochondria.

### 2.8. Measurement of Mitochondrial Membrane Potential

Cells were seeded and transfected as described previously. Different groups of cells treated with or without rotenone (100 nM) were used in the experiment. Changes in mitochondrial membrane potential (ΔΨ*m*) were measured by rhodamine 123 using flow cytometry (Becton Dickinson, USA) as previously described [26]. In brief, cells were incubated with serum-free medium containing 5 *μ*M rhodamine 123 for 30 min at 37°C and then collected. The fluorescent intensity correlating with ΔΨ*m* as a function of mitochondria was recorded at 488 nm excitation and 525 nm emission wavelengths.

### 2.9. ROS Detection

Cells were digested with collagenase IV (Gibco), pelleted, and suspended in the medium containing 20 *μ*M DCF-DA. After 30 min of incubation, cells were centrifuged at 2,000 ×g for 10 min, resuspended in a fresh medium, and subjected to FACS analysis (Beckman-Coulter MoFLo XDP cell sorter). The results were analyzed with FlowJo 7.6 software for the mean fluorescence intensity (MFI). The values are displayed relative to those obtained in solvent-treated control.

### 2.10. Annexin V and PI Double Staining Experiment

Cells were washed with PBS before resuspension in binding buffer. Double staining was performed with 10 *μ*L of Annexin V-FITC and 5 *μ*L of PI added to the resuspended cells. After incubation at room temperature for 15 min in the dark, 400 *μ*L of binding buffer was added to the cell suspension. Measurement of the cell sample was performed by flow cytometry (EPICS ALTRA, Beckman-Coulter, Miami, US) [27].

### 2.11. Statistics

All experiments were repeated three times. The data was presented as mean ± SEM and analyzed by ImageJ and SPSS software (version 16.0, IBM, Chicago, USA). The statistical significance of the differences among the groups was estimated by paired Student's* t*-test and/or one-way ANOVA, with 0.05 as the level of significance.

## 3. Results

### 3.1. BAG5 Interacts with DJ-1

**Figure 1:**
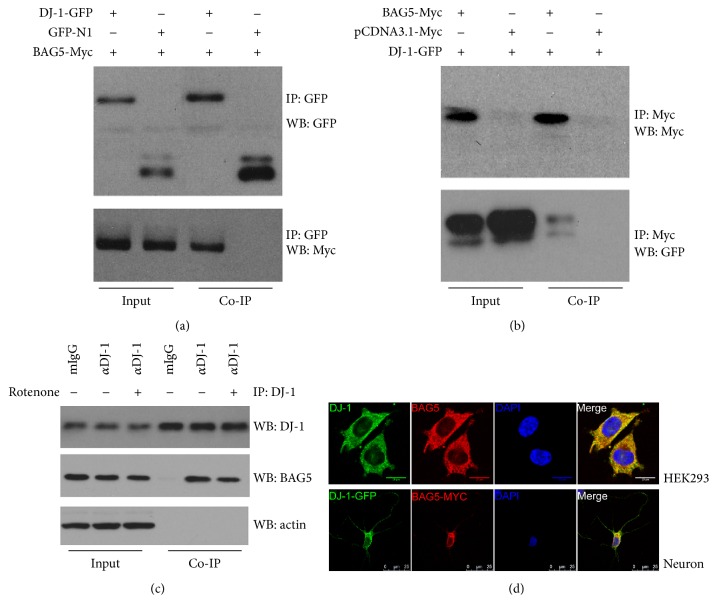
Interaction of BAG5 with DJ-1. (a) Co-IP of overexpressed DJ-1 and BAG5. HEK293 cells were cotransfected with DJ-1-eGFP and BAG5-Myc or eGFP followed by IP with anti-GFP polyclonal antibody. In Western blot, anti-GFP antibody (Ab1218) was used for examining DJ-1 and anti-Myc antibody (#2276) for BAG5. The results showed that DJ-1 could coimmunoprecipitate BAG5. (b) Conversely, similar results were obtained by anti-Myc antibody IP (#2272). (c) Co-IP was performed using DJ-1 antibody (*α*DJ-1, #5933) that recognized endogenous DJ-1. In Western blot, anti-DJ-1 antibody (ab11251) was used for examining endogenous DJ-1 and anti-BAG5 antibody (ab56738) for endogenous BAG5 protein. (d) Immunofluorescence colocalization analysis of DJ-1 (green) with BAG5 (red) in HEK293 cells (above) or primary hippocampal neurons of rat transfected with DJ-1-GFP (green) and BAG5-Myc (red) (below).

To investigate whether BAG5 interacts with DJ-1, we performed Co-IP assay in HEK293 cells. The full length of human BAG5 cDNA and DJ-1 cDNA was cloned. HEK293 cells were cotransfected with GFP-tagged DJ-1 and Myc-tagged BAG5 or EGFP vector followed by immunoprecipitation. The results showed that Myc-tagged BAG5 coimmunoprecipitated GFP-tagged DJ-1 (Figure 1(a)). Conversely, immunoprecipitation of DJ-1 brought down BAG5 (Figure 1(b)). Moreover, immunoprecipitation of endogenous DJ-1 coprecipitated BAG5 with or without rotenone (Figure 1(c)).

To further confirm the subcellular localization of DJ-1 and BAG5, immunofluorescence colocalization analysis was performed. DJ-1-GFP and BAG5-Myc plasmids were cotransfected in HEK293 cells or rat primary hippocampal neurons. Appropriate antibodies were used to detect the corresponding protein. Under the laser confocal microscope, both BAG5 (red) and DJ-1 (green) showed diffuse distribution in the cytoplasm. Moreover, merge images exhibited that BAG5 colocalized with DJ-1 in cytoplasm (Figure 1(d)).

### 3.2. BAG5 Attenuates the Stability of DJ-1

**Figure 2:**
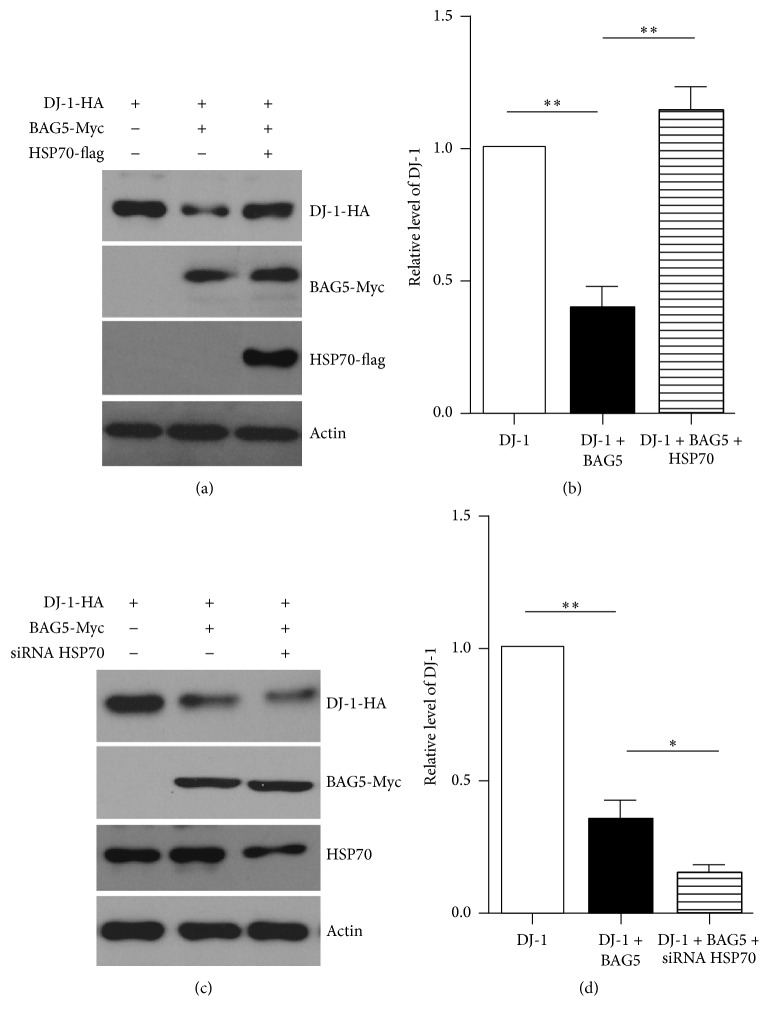
BAG5 reduces the levels of DJ-1, which are reversed by overexpressing Hsp70. (a) HEK293 cells stably expressing DJ-1-HA alone or with BAG5-Myc. Exogenous DJ-1 levels are obviously reduced in cells expressing BAG5-Myc comparing with cell expressing DJ-1-HA alone. Moreover, the overexpression of Hsp70-flag reversed the BAG5-mediated decrease of DJ-1-HA levels in cells stably expressing exogenous BAG5 and DJ-1. Actin was used as a loading control. (b) Quantitative data from (a) is shown. Data is normalized to DJ-1 group which was set to 1. (c) Knockdown endogenous Hsp70 accelerate reduction of ectopic DJ-1 induced by overexpression of BAG5. (d) Quantitative data from (c) is shown. The results are indicated as mean ± SEM (*n* = 3, ^*∗∗*^*p* < 0.01, ^*∗*^*p* < 0.05).

Since BAG5 interacts with DJ-1 in cultured cells, we next examined whether BAG5 can regulate the stability of DJ-1. HEK293 cells were stably expressing exogenous BAG5-Myc and DJ-1-HA or DJ-1-HA alone. Comparing with the latter, exogenous DJ-1 levels were obviously reduced in cells expressing BAG5-Myc. We further transfected Hsp70-flag in HEK293 cells stably expressing exogenous BAG5 and DJ-1. Interestingly, the overexpressing Hsp70 reversed the BAG5-mediated decrease of DJ-1 levels (Figures 2(a) and 2(b)). Consistently, with knockdown of endogenous Hsp70, Hsp70 deficiency can accelerate the reduction of ectopic DJ-1 induced by overexpression of BAG5 (Figures 2(c) and 2(d)).

**Figure 3:**
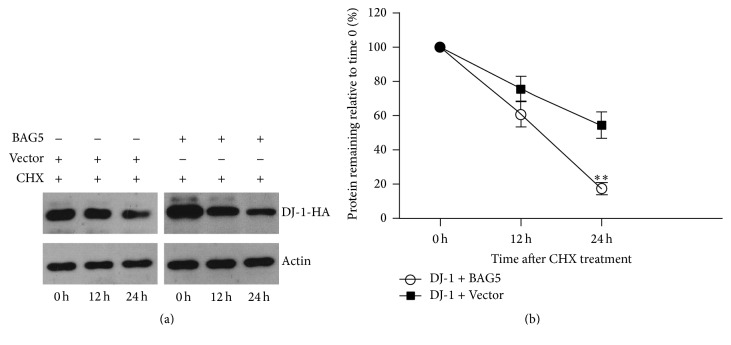
BAG5 attenuated the stability of DJ-1. (a) HEK293 cells stably expressing DJ-1-HA alone, or with BAG5-Myc. All cells were treated with 1 *μ*M CHX to inhibit total protein synthesis and cell's extracts were analyzed by Western blot using the specified antibodies at 0 hr, 12 hr, and 24 hr, respectively. Actin acted as a loading control. In the presence of CHX, BAG5 decreased the DJ-1 levels. (b) Quantitative data from (a) showed that it reduces half-life from 24 hr to 15 hr. Data is normalized to 0 h group which was set to 100%. The results are indicated as mean ± SEM (*n* = 3, ^*∗∗*^*p* < 0.01).

We further investigated whether BAG5 regulates the degradation of exogenous DJ-1. HEK293 cells expressing DJ-1-HA and BAG5-Myc or DJ-1-HA alone were treated with 1 *μ*M cycloheximide (CHX) to inhibit total protein synthesis followed by detecting the levels of DJ-1 protein at 0 hr, 12 hr, and 24 hr, respectively. HEK293 cells expressing DJ-1-HA and BAG5-Myc exhibited an increased degradation of exogenous DJ-1 protein after CHX treatment in comparison to cells expressing DJ-1-HA alone (Figure 3(a)). Quantitative data showed reduction in half-life from 24 hr to 15 hr (Figure 3(b)).

### 3.3. BAG5 Inhibits the Neuroprotective Effects of DJ-1 to Combat Mitochondrial Oxidative Damage

**Figure 4:**
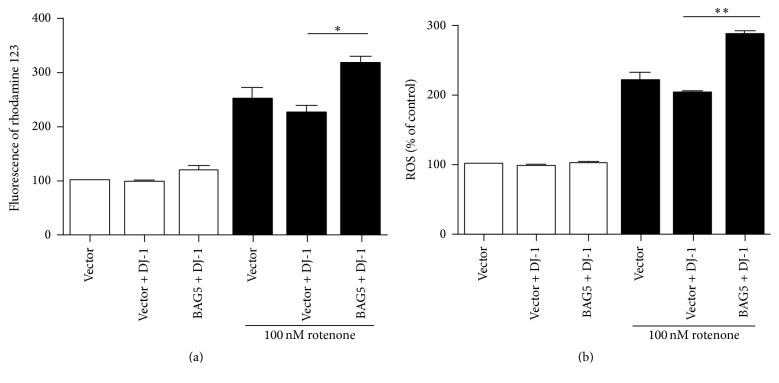
BAG5 attenuated the protective property of DJ-1 on mitochondrial function. HEK293 cells were stably expressed with empty vector or BAG5-Myc together with DJ-1-HA, with or without rotenone treatment. 24 h after treatment, ΔΨ*m* and ROS production were measured by flow cytometry. Quantitative analysis of the ΔΨ*m* and ROS was shown in (a) and (b), respectively. Data is normalized to vector + DJ-1 group which was set to 100%. Results are shown as the mean ± SEM (*n* = 3, ^*∗*^*p* < 0.05, ^*∗∗*^*p* < 0.01).

It is suggested that the loss of DJ-1 protein and homeostasis leads to mitochondrial defects. To identify whether BAG5 affects the protective property of DJ-1 in mitochondrial function, HEK293 cells expressing BAG5-Myc or BAG5-Myc with DJ-1-HA were treated with or without rotenone (100 nM) for 24 h. The changes of ΔΨ*m* and ROS production were measured by flow cytometry. Under rotenone, ΔΨ*m* decreased. However, coexpression of BAG5 and DJ-1 significantly reduced ΔΨ*m* even further (Figure 4(a)). Meanwhile, ROS level was significantly increased upon coexpression of BAG5 and DJ-1 compared with DJ-1 expression alone (Figure 4(b)).

**Figure 5:**
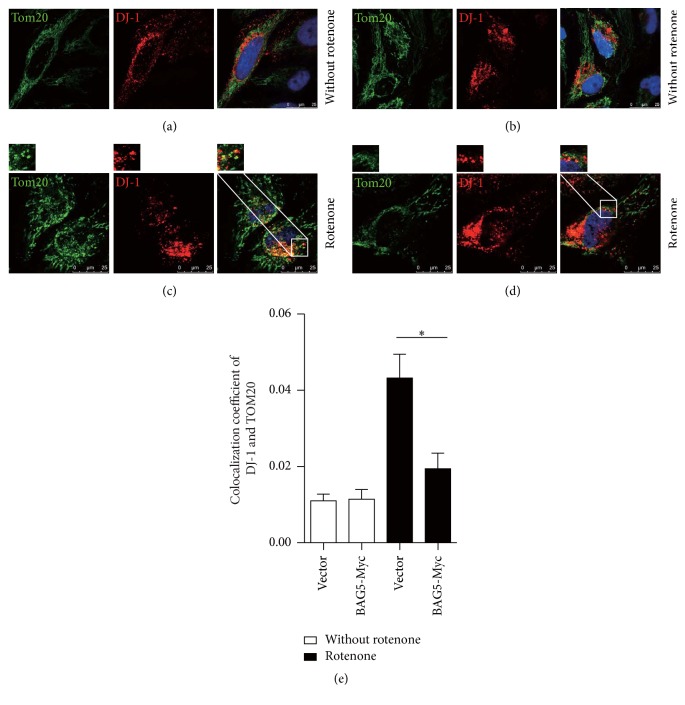
BAG5 inhibited DJ-1 recruitment to mitochondria under rotenone treatment. HEK293 cells stably expressed DJ-1-HA alone (a, c) or with BAG5-Myc (b, d). DJ-1 and mitochondria were stained by HA (red) and Tom20 (green) antibodies, respectively. Without rotenone treatment, exogenous DJ-1-HA (green) has almost no localization on mitochondria marker Tom20 (red) in cells stably expressing vector (a) or BAG5-Myc (b). After being treated with rotenone, increased mitochondrial localization of DJ-1 was observed in cells stably expressing DJ-1-HA alone (c) comparing with BAG5-Myc + DJ-1-HA (d). The enlarged area is displayed as a white box. Quantitative analysis of colocalization is shown (e). Results are shown as mean ± SEM (^*∗*^*p* < 0.05).

Further, immunofluorescence test showed that, without rotenone treatment, exogenous DJ-1-HA has almost no localization with mitochondria in cells stably expressing vector (Figure 5(a)) or BAG5-Myc (Figure 5(b)). After treating with rotenone, DJ-1-HA was diffusely distributed in mitochondria (Figure 5(c)). But the levels of DJ-1-HA distribution in mitochondria were significantly decreased in cells having stable expression of BAG5-Myc and DJ-1-HA in the presence of rotenone (Figure 5(d)). Quantitative data is shown in Figure 5(e).

### 3.4. BAG5 Reduces DJ-1 Dimerization

**Figure 6:**
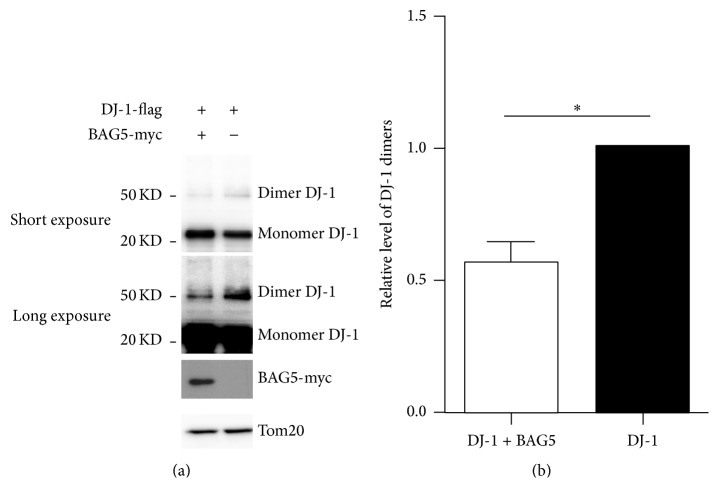
BAG5 influences the dimerization of DJ-1 in mitochondria. Mitochondria were isolated in HEK293 cells stably expressing DJ-1-flag alone or with BAG5-Myc for examining the levels of dimer and monomer by Western blot. Tom20 acted as control protein in mitochondria. HEK293 cells coexpressing BAG5 and DJ-1 had a lower level of dimer and a higher level of monomer in mitochondria than HEK293 cells expressing DJ-1 alone (a). Quantitative data from (a) is shown (b). Results were shown as the mean ± SEM (*n* = 3, ^*∗*^*p* < 0.05).

Localization of DJ-1 as a dimer in mitochondria is required for its function in antioxidative stress reaction [28]. Therefore, we speculated that the cause of BAG5 decreasing ΔΨ*m* and DJ-1 levels in mitochondria is its effect on dimerization of DJ-1. To confirm this, mitochondria were isolated from HEK293 cells stably expressing DJ-1 with or without BAG5-Myc followed to detect the levels of dimer and monomer by Western blot. Comparing with HEK293 cells expressing DJ-1 alone, HEK293 cells coexpressing BAG5 and DJ-1 had a lower level of dimer and a higher level of monomer in mitochondria (Figure 6).

**Figure 7:**
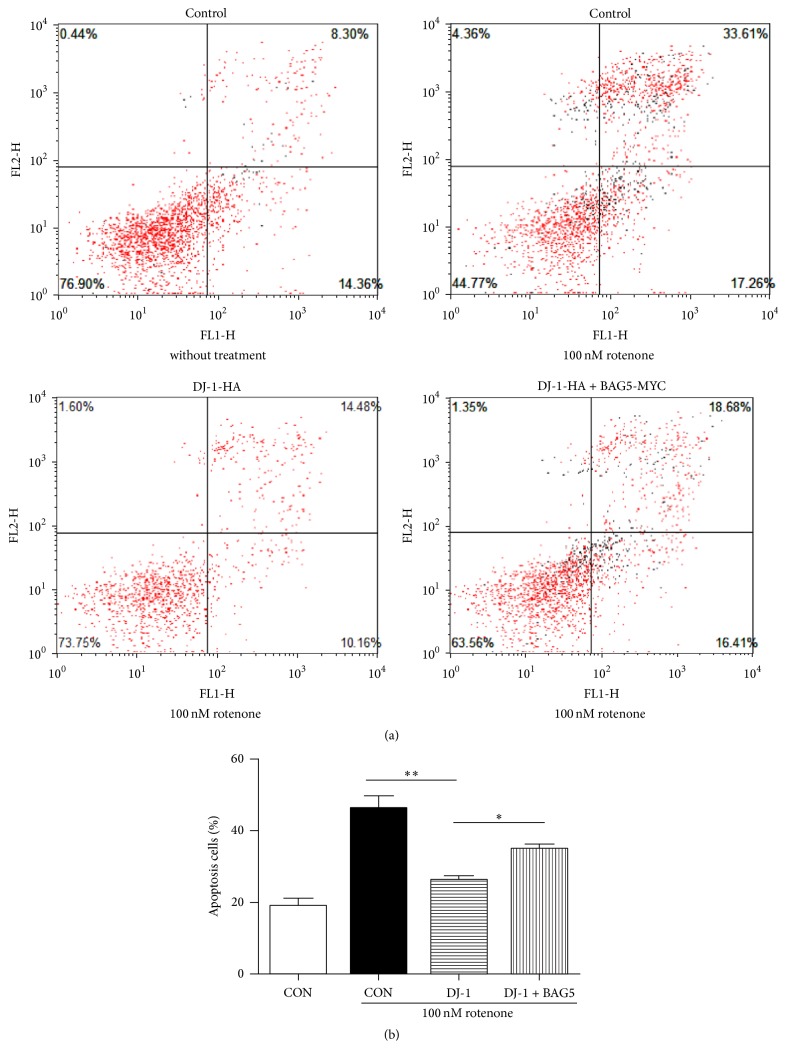
BAG5 diminished the protective effects of DJ-1 on rotenone-induced cell apoptosis. The stable expression of cells is indicated (a). After 24 h of rotenone treatment, the induction of apoptosis was determined by Annexin V-FITC/PI double staining assay, and the degree of apoptotic cell death was quantified. Quantitative data from (a) is shown in (b). Results are shown as mean ± SEM (*n* = 3, ^*∗*^*p* < 0.05, ^*∗∗*^*p* < 0.01).

Moreover, Annexin V and PI double staining were performed followed by flow cytometry to detect apoptotic cells. Consistence with the reported data [29], we showed that overexpression of DJ-1 significantly attenuated the rotenone-induced apoptosis. However, HEK293 cells coexpressing DJ-1 and BAG5 showed increased apoptosis compared to the cells expressing DJ-1 only (Figure 7).

## 4. Discussion

After Parkin, DJ-1 is the second cloned pathogenic gene involved in autosomal recessive early-onset Parkinsonism. It has been suggested that the interactions of DJ-1 and some other proteins may play an important role in the process that DJ-1 protects cells against oxidative stress and maintains mitochondrial homeostasis [13, 14]. Therefore, to identify the pathogenic mechanism of DJ-1, researchers increasingly focus on looking for other proteins which interact with it.

Chaperons and cochaperones, including BAG family, Hsp70, and CHIP, are closely associated with the ubiquitin-proteasome system (UPS) and the autophagy-lysosomal pathway, that are the hallmark of protein degradation [30]. Recently, studies on the relationship of BAG family and PD-related proteins have shown positive findings, strengthening the link between the BAG proteins and PD-related neurodegeneration [31, 32]. With Co-IP and immunofluorescence experiments, we demonstrate that BAG5 interacts with DJ-1. Furthermore, overexpressing BAG5 led to the decrease in exogenous DJ-1 levels, which was reversed by Hsp70. It revealed that Hsp70 and BAG5 regulate the stability of DJ-1.

However, it has been reported that DJ-1 is not significantly aggregated after treating with proteasome inhibitor MG132, indicating that most of DJ-1 is not ubiquitinated and degraded via proteasome pathway [14, 33]. Studies on crystal structure of DJ-1 proved that DJ-1 presents as a dimer [34, 35]. In addition, the disease-related mutation DJ-1 L166P shifts its subcellular distribution to the nucleus and decreases its ability to dimerize, whereas BAG1, the first discovered BAG family protein, restores DJ-1 L166P subcellular distribution and dimerization [36]. This indicates that, apart from enhancing the degradation of DJ-1 through pathways other than UPS, BAG5 may reduce the stability of DJ-1 by shifting its subcellular distribution and affecting its dimer formation.

Under normal condition, only a small fraction of DJ-1 is located in the mitochondria and regulates its activity by binding to subunits of mitochondrial complex I. When exposed to oxidative stress, mounts of DJ-1 homodimers translocate to mitochondria with the help of molecular chaperones thus preventing oxidative stress-induced cell death [37, 38]. This study suggests that wild-type DJ-1 protected against rotenone-induced cell apoptosis and mitochondrial damage, and this process could be abolished by BAG5. Moreover, our findings demonstrate that BAG5 reduces the level of DJ-1 dimers in mitochondria, which suggests that BAG5 inhibits dimerization and mitochondrial translocation of DJ-1. In particular, overexpression of Hsp70 could partly reverse retinal degeneration and necrosis reduced by knocking down of DJ-1a in Drosophila (results were not given), suggesting that Hsp70 and BAG5 exerted opposite effects on neuroprotective function of DJ-1.

Rotenone causes mitochondrial dysfunction. Damaged mitochondrial fragments induce apoptosis by the release of proapoptotic proteins or are removed by mitophagy [39–41]. Mitophagy is a major process for mitochondrial quality control to maintain mitochondrial homeostasis and protect against oxidative stress [42]. Studies suggest that PINK1 plays important roles in mitophagy [43, 44]. Recently, Wang et al. [21] have reported that BAG5 rescues oxidative stress-induced mitochondrial dysfunction by upregulating PINK1 via ubiquitin-proteasome system. BAG5 may enhance mitophagy to protect mitochondria by stabilizing PINK1. But in our case, BAG5 decreased stability of DJ-1 and weakened its role in mitochondrial protection. We prove that BAG5 reduces DJ-1 dimerization conferring hypersensitivity to oxidative stress. So, there is need to maintain a balance between cell apoptosis induced by mitochondrial oxidative stress and eliminating damaged mitochondria by mitophagy to protect against apoptosis.

In conclusion, we demonstrate that BAG5 interacts with PD-related DJ-1 and it negatively regulates the dimerization of DJ-1, attenuating the DJ-1-mediated protection of mitochondria and cell survival under oxidative stress. Thus, it is conceivable that BAG5, by affecting DJ-1 stability, would significantly contribute to PD pathogenesis. These findings may have important implications for understanding the pathogenesis of PD and ultimately novel therapeutic.
